# Resin Diterpenes from *Austrocedrus chilensis*

**DOI:** 10.3390/molecules161210653

**Published:** 2011-12-20

**Authors:** Verónica Rachel Olate, Olatz Goikoetxeaundia Usandizaga, Guillermo Schmeda-Hirschmann

**Affiliations:** 1 Laboratorio de Química de Productos Naturales, Instituto de Química de Recursos Naturales, Universidad de Talca, Casilla 747, Talca, Chile; 2 Facultad de Ciencias Químicas, Campus de Gipuzkoa, Euskal Herriko Unibertsitatea, Pº Manuel de Lardizábal 3, 20018 Donostia-San Sebastián, Spain

**Keywords:** *Austrocedrus chilensis*, diterpenes, labdanes, abietanes, Andean gymnosperms

## Abstract

Seventeen diterpenes belonging to the labdane, abietane and isopimarane skeleton classes were isolated from the resin of the Chilean gymnosperm *Austrocedrus chilensis* and identified by spectroscopic and spectrometric methods. The diterpene 12-oxo-labda-8(17),13*E*-dien-19 oic acid is reported for the first time as a natural product and 14 diterpenes are reported for the first time for the species.

## 1. Introduction

The gymnosperm tree *Austrocedrus chilensis* (D. Don) Florin et Boutelje (Syn.: *Austrocedrus chilensis* (D.Don) Pic. Ser. et Bizz) (Cupressaceae) is known as “ciprés de cordillera” and grows in sandy soils in the Eastern Andean slopes up to 2,000 m over sea level. The plant leaves has been used as a sudorific and the powdered fruits to treat diarrhea [1]. Little information exists on other medicinal uses of the plant, but the wood is highly appreciated. The monoterpene carvacrol and β-thujaplicin (4-isopropyl tropolone = hinokitiol) and the flavonoid taxifolin were isolated from the wood of *A*. *chilensis* [2]. An ethanol extract from the aerial parts of the tree, including leaves, twigs and stems, afforded the lignan desoxypodophyllotoxin and the diterpenes 8,20-dihydroxy-8(11),13-abietadien-12-one and pisiferol [3,4,5]. Flavonoids were identified from a 70% ethanol extract of leaves and branchlets of *A. chilensis* [6], while sugiol and another unidentified diterpene were isolated from a tree bark methanol extract [7].

However, scarce information is available on the composition of its resin. Cox *et al.* [8], working on a comparison of gymnosperm resins using hyphenated techniques, described the identification of two labdane/clerodane C-19 acids, sandaracopimaric acid, ferruginol, 2,3-dehydroferruginol, 6,7-dehydro-ferruginol and 7-oxoferruginol from the external resin of *A. chilensis*.

The investigation of gymnosperm resin composition has been carried out using chromatographic means for the isolation and further identification of the constituents by spectroscopic and spectrometric methods [9,10,11]. New techniques used to characterize the resin constituents include gas chromatography coupled to mass spectrometry (GC-MS) [12,13,14,15], and proton magnetic resonance spectroscopy [16].

Following our studies on native South American plants, we have now examined the composition of resin exudates from a mature population of *Austrocedrus chilensis* trees to establish suitable conditions for metabolic profiling.

## 2. Results and Discussion

Seventeen diterpenes with isopimarane, labdane and abietane skeletons were isolated from the resin of *Austrocedrus chilensis*, and identified by spectroscopic and spectrometric methods. Fourteen of these diterpenes are reported for the species for the first time. The compounds were identified by spectroscopic means and by comparison of their spectral data with literature values. The ^13^C-NMR data of compounds **4**, **6**, **12–14a** is shown in Table 1.

**Table 1:** ^13^C-NMR data of the compounds **4**, **6**, **12**, **13** and **14a** (100 MHz, CDCl_3_, δ-values).

C	4	6	12	13	14a
1	36.3 t	41.7 t	39. 5 t	39.5 t	39.5 t
2	18.9 t	19.6 t	26.0 t	26.0 t	26.0 t
3	35.2 t	38.6 t	38.1 t	38.1 t	38.2 t
4	37.9 s	40.1 s	44.4 s	44.4 s	44.5 s
5	45.5 d	55.8 d	56.5 d	56.5 d	56.2 d
6	21.4 t	24.7 t	20.1 t	20.1 t	20.1 t
7	35.3 t	39.3 t	38.7 t	38.7 t	38.4 t
8	124.5 s	148.9 s	148.1 s	148.1 s	149.4 s
9	137.2 s	57.6 d	56.6 d	56.9 d	51.1 d
10	37.6 s	33.8 s	40.6 s	40.6 s	39.7 s
11	18.5 t	17.9 t	23.5 t	23.5 t	33.0 t
12	32.3 t	42.4 t	134.1 d	131.9 d	201.1 s
13	35.2 s	73.8 s	133.6 s	131.7 s	139.1 s
14	42.3 t	145.5 d	141.8 d	133.9 d	136.0 d
15	146.5 d	111.8 t	110.1 t	113.4 t	14.9 q
16	110.9 t	27.9 q	12.0 q	12.0 q	11.5 q
17	28.1 q	106. 7 t	107.9 t	108.0 t	106.3 t
18	72.5 t	21.9 q	29.2 q	29.2 q	29.0 q
19	17.7 q	33.8 q	184.4 s	184.4 s	178.0 s
20	20.1 q	14.7 q	13.0 q	13.0 q	13.3 q
OMe	-	-	-	-	51.4 q

**Figure 1:**
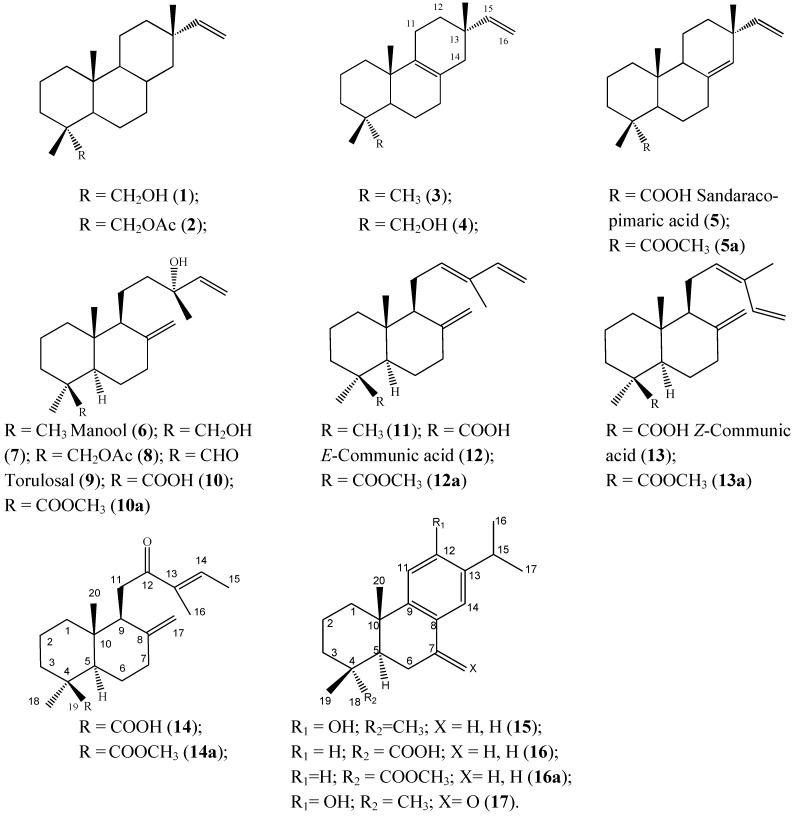
Structure of the compounds isolated and/or identified from the resin of *Austrocedrus chilensis*.

The structures of the compounds identified in the resin are shown in Figure 1. From the compounds identified in the present work, only sandaracopimaric acid (compound **5**), ferruginol (compound **15**), and 7-oxoferruginol (sugiol, compound **17**) were previously reported [8] in a resin sample of this species collected in southern Chile. On the other hand, the previously reported diterpenes 2,3-dehydroferruginol and 6,7-dehydroferruginol [8] were not identified in our samples. The differences can be explained due to different plant populations and collection time.

Related diterpenes were isolated from *Cryptomeria japonica*, including the labdanes imbricatolic acid, its 15-acetoxy derivative, imbricatolic acid methyl ester, labd-8(17)-en-15,19-dioic acid dimethyl ester, 15-(2-oxopropylidene)labd-8(17)-en-19-oic acid, 13-epimanool, 13-epitorulosol, 19-acetoxy-13S-hydroxylabda-8(19),14-diene, 13-epitorulosal, 13-epicupressic acid, agathadiol, 15-acetyl-agathadiol, 19-acetylagathadiol, 15,19-diacetylagathadiol, isoagatholal, 15-acetylisoagatholal, iso-cupressic acid, 15-acetylisocupressic acid, 15-oxolabda-8(17),13*E*-dien-19-oic acid, 7β-acetoxy-15-hydroxylabda-8(17), 13*E*-dien-19 oic acid methyl ester, 14-hydroxy-15-norlabd-8(17)-en-19-oic acid methyl ester, 15-hydroxylabda-8(17),13*Z*-dien-19 oic acid methyl ester, 15,16-epoxylabda-13(16),14-dien-8α,19-diol, 15-acetoxylabda-8,13*E*-dien-19 oic acid, 8α-hydroxylabda-13(16),14-dien-19-yl *p*-methoxycinnamate, cryptomeridiol-4-yl-19-acetoxylabda-8(19),13*E*-dien-19-oate [17]. In a new article, additional labdanes, abietanes and pimaranes were identified, including junicedric acid, 13-epi-cupressic acid methyl ester, copalol, 13-oxo-14,15-dinorlabd-8(17)-en-19 oic acid methyl ester, *trans*-communic acid, *cis*-communic acid, 19-acetoxyferruginol, sugiol methyl ether, 6α-hydroxydemethyl-cryptojaponol, 5,6-dehydrosugiol methyl ether, cupresol, nejukol, isopimarinol and isopimaric acid [18].

The compound **14** was isolated as the corresponding methyl ester and is described as a new natural product. The structure of compound **14** follows from the HR-MS indicating a molecular formula C_20_H_30_O_3_ and C_21_H_32_O_3_ after methylation (compound **14a**), accounting for six degrees of unsaturation (*i.e.*, three double bonds, two rings and one carbonyl function). The IR spectrum of the methyl ester shows an ester function and α,β-unsaturated ketone at 1,724 and 1,672 cm^−1^, respectively. In the ^1^H-NMR spectrum, an olefinic side chain proton at δ 6.77 (H-14) coupled with two allyl methyl groups at δ 1.84 (H-15) and 1.74 ppm (H-16), indicates an α,β-unsaturated ketone system in the side chain of the diterpene. The *E*-configuration of the side chain double bond follows from the chemical shift of the olefinic proton and the C-15 methyl group and is in agreement with the data reported for related diterpenes isolated from the liverwort *Scapania undulata* [19].

The pair of dd at δ 2.94 (17.5, 10.9) and δ 2.50 (17.5, 3) ppm (H-11) places the ketone at C-12. An exo methylene (δ 4.67, s, H-17 and δ 4.23, s, H-17′), a methoxy group at δ 3.60 and two quaternary methyl groups appears as singlets at δ 1.18 (H-18) and 0.54 ppm (H-20), respectively, indicating that the diterpene has a labdane skeleton. The ^1^H-NMR data of compound **14** are summarized in the Experimental section. The ^13^C-NMR spectrum is in agreement with the proposed structure. Related compounds with a hydroxy function at C-15 were reported from *Chloranthus henryi* including henrilabdane A and the 13-oxo derivative henrilabdane C [20] and 12,13RS-dihydroxycommunic acid from *Platycladus orientalis* [21].

**Figure 2:**
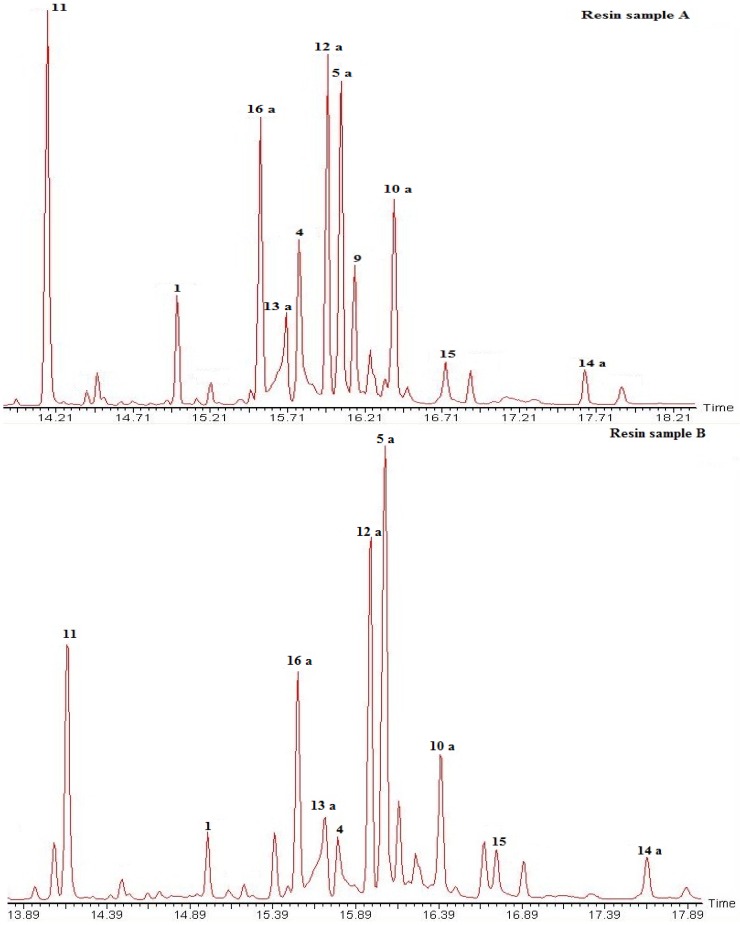
GC trace of the *Austrocedrus chilensis* resin (as methyl esters). Compounds were identified by comparison with standards isolated from the resin and comparison of the mass fragmentation patterns with literature. Compounds: **1**: 18-hydroxy isopimar-15-ene; **4**: Isopimara-8(9),15-dien-19-ol; **5a**: Sandaracopimaric acid methyl ester; **9**: Torulosal; **10a**: Torulosic acid methyl ester; **11**: 8(17),12,14-labdatriene; **12a**: *E*-communic acid methyl ester; **13a**: *Z*-communic acid methyl ester; **14a**: 12-oxo-labda-8(17),13*E*-dien-19 oic acid methyl ester; **15**: Ferruginol; **16a**: Dehydroabietic acid methyl ester. For compounds **6**, **9** and **10**, M^+^-water was detected instead of the molecular ion. The resin sample A was obtained from a female tree while the sample B was taken from a male tree from the same population. Both samples were collected in November 2010. For compound structures please see Figure 1.

The methylated resin samples (200–240 mg) from a female (A) and a male tree (B) were separately loaded on preparative TLC plates (SiO_2_, PE-EtOAc 85:15). After visualization under UV light, the different elution bands were comparatively analyzed by ^1^H-NMR and GC-MS. The GC traces of the resin samples is shown in Figure 2. Fraction 1 (Rf 0.75) showed a mixture of compounds **5a**, **12a** and **13a**. Fraction 2 (Rf 0.62–0.70) contained compounds **1**, **5a**, **12a**, **13a**, **15** and **16a**. Fraction 3 (Rf 0.50–0.62) yielded compounds **11**, **15** and **14a**. Fraction 4 showed a main spot (Rf 0.38) and a second spot (Rf 0.50), the constituents were identified as compounds **6** and **4**. Fraction 5 contained a minor constituent with Rf 0.38 and a main spot with Rf 0.30 identified as compounds **9**, **10a** and **4** as minor constituents.

The compounds isolated and identified from the *A. chilensis* resin are constituents occurring in several gymnosperms and in the Cupressaceae family. The C-13 epimer of compound **3** was previously described in a study of cationic rearrangements and cyclizations of diterpenes [22] as well as from *Phoma betae* [23]. Compound **4** was identified as 8,15-isopimaradien-19-ol and shows clear differences with the *ent*-isopimaranes isolated from *Calceolaria polifolia* [24] and *C. latifolia* [25]. Sandaracopimaric acid (**5**) as well as several abietanes were isolated from the endemic species *Taxus mairei* occurring in Taiwan [26], *Juniperus chinensis* [27] and other gymnosperms [5]. 8(17),14-Labdadien-13-ol (**6**) was previously reported from *Cupressus sempervirens*, *C. torulosa*, *Tetraclinis articulate*, *Vitex rotundifolia* [5], *Cupressus dupreziana* and *C. sempervirens* [11]. The ^13^C-NMR data of compound **6** agree with that reported for epimanool derivatives [17]. Compounds **6–10** were described as constituents of *Cryptomeria japonica* leaves [18]. Torulosal (**9**) has also been previously isolated from *Cupressus torulosa*, *Araucaria cooki*, *Tetraclinis articulate* and *Dacrydium biforme* [5]. The communic acid isomers (compounds **12** and **13**) has been reported from several Cupressaceae, including *Juniperus chinensis* [27] and ferruginol (**15**) has been found in several Podocarpaceae and gymnosperms [28]. Dehydroabietic acid **16** is a common constituent in resins from gymnosperms and 7-oxoferruginol (sugiol, compound **17**) was identified [8] from a Chilean resin sample of *A. chilensis*.

Overall, the diterpene composition of the South American Cupressaceae *Austrocedrus chilensis* shares similarities with *Juniperus* and *Cryptomeria* species, including the occurrence of *E*- and *Z*-communic acid, sandaracopimaric acid, ferruginol and several labdane and pimarane diterpenes [27,17]. Labdane diterpenes have been reported from other Cupressaceae including *Platycladus orientalis* [29], while *Juniperus brevifolia* afforded sandaracopimaranes and abietanes [30]. *Juniperus phoenicea* and *J. thurifera* var. *africana* yielded several abietane and pimarane derivatives [31]. Labdanes and pimarane acids have been isolated from *Juniperus thurifera* [9,10].

Several of the diterpenes occurring in the *A. chilensis* resin have been found to display relevant biological activities in different *in vitro* as well as in *in vivo* systems. Communic acid has been reported as a selective COX-2 enzyme inhibitor from *Curcuma mangga* [32] and antimycobacterial compound in *Juniperus communis* [33]. Ferruginol present gastroprotective and cytotoxic activity [34,35], antiproliferative [36], anti-staphylococcal [37] and antiplasmodial effect [38], among others. The related diterpene 7-oxoferruginol was associated with the anti-inflammatory and hepatoprotective effect of *Cryptomeria japonica* [39]. Dehydroabietic acid and its derivatives present gastroprotective effect in animal models of induced gastric ulcers [40]. The abietane diterpenes 20-hydroxyferruginol and 6α-hydroxysugiol from the cones of *Sequoia sempervirens*, have been shown to present antitumour effect in oncogenic H-*ras* transformed cells and also against the following human tumor cell lines: colon (SW620 and HCT116), breast (MDA-MB-231) and lung (NCI-H23 and A 549) [41]. Labdane diterpenes from *Thuja standishii* stem bark including 6α-hydroxysugiol, 12S-hydroxylabda-8(17), 13(16),14-trien-19-oic acid, 12-methoxyabieta-8,11,13-trien-11-ol and 15-oxolabda-8(17),11*Z*,13*E*-trien-19 oic acid displayed inhibitory effects as potential cancer chemopreventive agents [42].

While the composition of resin samples from a female and a male tree showed the same main compounds, the relative ratio of the diterpenes was different. This fact can be related to several factors, including gender, individual, seasonal, or response to pathogens, among others. However, a much larger number of samples should be analyzed to disclose the significance of the present findings. The establishment of a chromatographic method for the fast and reliable identification of the resin constituents opens new possibilities for the comparative study of populations of this tree as well as the response of *A. chilensis* to environmental stress and microorganisms [43,44,45,46], including the pathogenic fungus *Phytophthora austrocedrae*.

## 3. Experimental

### 3.1. General

Optical rotations were obtained for solutions in CHCl_3_ (concentrations expressed in g/100 mL) on a Jasco DIP 370 polarimeter (Jasco Analytical Instruments, Easton, MD, USA). IR spectra were recorded on a Nicolet Nexus FT-IR instrument (Thermo Electron Corporation, Waltham, MA, USA). All NMR experiments were performed on a Bruker Avance 400 NMR spectrometer (Bruker BioSpin GmbH, Rheinstetten, Germany) equipped with a 5 mm inverse detection z-gradient probe. The ^1^H and ^13^C spectra (at 400 and 100 MHz, respectively) were measured at room temperature (22–23 °C) using CDCl_3_ as solvent. Chemical shifts are given on the δ scale and were referenced to residual CHCl_3_ at 7.25 ppm for ^1^H spectra and to the solvent at 77.00 ppm for ^13^C spectra. One-dimensional ^1^H and ^13^C-NMR spectra were acquired under standard conditions. The pulse programs of the COSY, gHSQC, gHMBC experiments were taken from the Bruker software library. Homonuclear two-dimensional spectra (COSY) and inverse proton-detected heteronuclear two-dimensional spectra (gHSQC) were acquired in the phase-sensitive mode and gHMBC spectra were acquired in the absolute value mode. The data for the gHSQC spectra were collected in a 1024 × 256 matrix with a spectral width of 4,000 Hz in the proton domain and 20,000 Hz in the carbon domain and processed in a 1024 × 512 matrix. The gHSQC experiments were optimized for a one-bond heteronuclear coupling constant of 145 Hz. The gHMBC experiments were optimized for long-range coupling constants of 7.96 Hz. 2D-NOESY experiments were acquired with a mixing time of 500 ms, a recycle delay of 1 s and 128–256 transients per spectrum. Coupling constants *J* values are presented in Hz. HR-MS were measured with an VG Autospec Trisector EBE (Micromass Instruments S.A., Madrid, Spain) spectrometer operating at 70 eV and are presented as *m/z* (rel. int. %). Silica gel 60 (Merck, 63–200 µm particle size) was used for column chromatography, precoated silica gel plates (Merck, Kieselgel 60 F_254_, 0.25 mm) were used for thin layer chromatography (TLC). TLC spots were visualized by spraying the chromatograms with *p*-anisaldehyde-ethanol-acetic acid-H_2_SO_4_ (2:170:20:10 v/v) and heating at 110 °C for 3 min.

### 3.2. GC-MS Analysis

Equipment: Perkin Elmer Turbo Mass (Perkin-Elmer Corporation, Norwalk, CT, USA). Column: fused silica capillary column, SP-2330 (Supelco), 30 m × 0.25 μm. Carrier: He, split flow 50.0 mL/min, initial setpoint: 20.0 PSIG. Oven program: total run time: 66 min, initial temperature: 100 °C, initial hold: 1.00 min, Ramp: 10.0 °C/min to 250 °C, hold for 50.00 min. Injection volumen: 1 μL. Compounds were characterized by electron-ionization (EI) mass spectra. Retention time (Rt, min) and MS fragmentation patterns of the known compounds were compared with literature.

### 3.3. Plant Material

The resin of *Austrocedrus chilensis* was collected from a mature tree population growing at Las Trancas, VIII Region, Chile (36°54′03′′S, 31°32′47′′W). Voucher herbarium specimens have been deposited at the Herbario de la Universidad de Talca and were identified by Patricio Peñailillo. The voucher specimens correspond to the individuals from those the resin was obtained. For the phytochemical study, resin was collected in February 2009 while for GC-MS analysis, samples were obtained on November 2010. For the composition analysis and isolation of the diterpene constituents, samples were randomly collected from adult trees of either gender. Naturally exuding resin drops were manually collected and stored in glass containers for analysis. For the GC-MS analyses, part of the resin was dissolved in diethyl ether and then treated with diazomethane in diethyl ether to obtain the corresponding methyl esters. The resin drop samples as collected were dissolved in CDCl_3_ for ^1^H-NMR analysis and comparison with the results obtained using GC-MS. Two resin samples (A and B) collected on November 01, 2010 were worked-up after methylation to compare the GC-MS patterns with the ^1^H-NMR spectra, allowing identity confirmation of all main constituents.

### 3.4. Isolation of the Resin Constituents

The crude resin (362 g) was extracted with a 1:1 dichloromethane (DCM)-ethyl acetate (EtOAc) mixture. Some 110.58 g of solubles were obtained. The resin was adsorbed on silica gel and submitted to flash chromatography with a petroleum ether (PE)/ethyl acetate (EtOAc) gradient (PE/EtOAc 95:5, 90:10, 80:20, 70:30, 60:40, 50:50 0:100). The volumen of each fraction was 1 L. After TLC analysis (SiO_2_, PE/DCM 1:1 and PE/EtOAc 9:1), fractions with similar patterns were pooled as follows: 1 (280 mg), 2 (20 mg), 3 (7 g), 4 (16 g), 5 (17.5 g), 6 (12 g), 7 (11 g) and 8 (15 g) (total: 78.8 g).

Fractions 1 and 2 containing mainly non-polar compounds were submitted to GC-MS. The GC-MS chromatogram of both fractions showed a main compound, identified as **3** according to the MS fragmentation pattern (NIST database) and interpretation of the spectra, with **4** as a second product. The GC-MS profile of fractions 3 and 4 presented a main constituent identified as 8(17), 12,14-labdatriene (**11**), the alcohol **1** and the compound **3**. Fractions 5, 6 and 7 differed only in the relative proportion of constituents. Part of the fraction 6 (12 g) was permeated on a Sephadex LH-20 column (column length 125 cm, Sephadex content 60 cm, i.d. 5 cm) with a PE/DCM/MeOH 1:1:1 mixture in two batches (2 × 6 g each). Some 32 fractions of 20 mL each were collected and pooled together according to the TLC patterns (SiO_2_, PE/EtOAc 8:2). Fractions 1–10 did not contained compounds of interest and were discarded. The pool of fractions 11–14 (1.67 g) was chromatographed on a silica gel column (190 g silica gel, 70 cm length, 4 cm i.d.) with a PE/EtOAc 8:2 mixture. Some 44 fractions of 25 mL each were collected and grouped after TLC analysis as follows: 1–2 (discarded), 3–5 (319 mg) with manool (**6**) as the main constituent and the acetate **2** as a minor compound, 6–7 (42 mg manool **6**), 8–9 (32 mg isopimara-8(9),15-dien-19-ol, **4**), 10–21 (350 mg) and 22–43 (638 mg, complex mixture). The fraction pool 10–21 afforded after preparative TLC on silica gel (toluene/EtOAc 9:1) 64 mg of a mixture of 18-acetoxymanool (**8**) and torulosal (**9**).

The fraction pool 15–19 (1.92 g) was chromatographed on a RP-8 silica gel column with MeOH/H_2_O 9:1 as the mobile phase in three batches. Some 90 fractions of 5 mL each were collected and pooled after TLC to afford a mixture of *E*-communic acid (main compound) and *Z*-communic acid (compounds **12** and **13**) in a 3:1 ratio (350 mg) and sandaracopimaric acid (90 mg, **5**). A representative sample of the fractions 1–32 (136 mg) was treated with diazomethane. After preparative TLC (silica gel, PE/EtOAc/acetone 9:2:1) 51 mg of torulosic acid methyl ester (**10a**) and 33 mg of a mixture of torulosic acid methyl ester (**10a**) and the alcohol **7** were obtained. After methylation, preparative TLC of fraction pool 20–23 (toluene/DCM/Et_2_O 4.5:4.5:1) yielded 12.3 mg of compound **14a** and 13 mg of a mixture of the compounds **6** and **7** in a 4:1 ratio.

**Figure 3:**
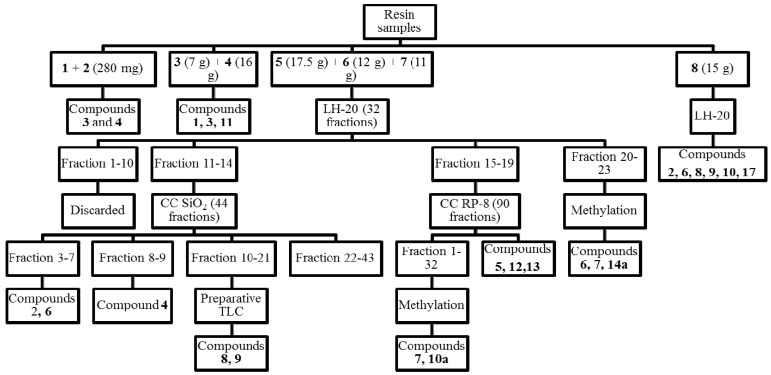
Flow chart summarizing the isolation of constituents from *Austrocedrus chilensis* resin.

Some 4.06 g of fraction 8 were permeated on a Sephadex LH-20 column (125 cm length, 5 cm i.d., 60 cm Sephadex filling), with a PE:DCM:MeOH 1:1:1 mixture. Some 34 fractions of 20 mL each were collected and pooled according to the TLC patterns to afford after additional CC on silica gel, 10 mg of the acetate **2** with traces of the corresponding aldehyde, 2.5 mg manool (**6**) and 15.6 mg of the acetate **8** with traces of torulosal (**9**), torulosic acid (**10**, 362 mg) and traces of 7-oxoferruginol (sugiol, **17**). The isolation of the compounds is summarized in Figure 3.

### 3.5. Compound Characterization

*18-Hydroxyisopimar-15-ene* (**1**). GC-MS: Rt 14.98 min; C_20_H_34_O (290); *m/z* 290 (11), 275 (34), 257 (40), 179 (20), 137 (41), 109 (65), 95 (66), 81 (79), 69 (84), 55 (100).

*18-Acetoxyisopimar-15-ene* (**2**). [α]_D_^20^: +82.7 (*c* 0.10, CHCl_3_); IR *v_max_* (film) 2928, 2864, 1736, 1461, 1378, 1242. GC-MS: Rt 16.33 min; C_22_H_34_O_2_ (330); *m/z* 330 (9), 315 (7), 288 (6), 270 (9), 255 (70), 187 (30), 105 (47), 91 (53), 43 (100).

*Isopimara-8(9),15-diene* (**3**). GC-MS: Rt 14.13 min; C_20_H_32_ (272); *m/z* 272 (11), 257 (34), 137 (53), 107 (53), 95 (89), 81 (100), 69 (91), 55 (89).

*Isopimara-8(9),15-dien-19-ol* (**4**). [α]_D_^20^: +58.8 (*c* 0.32, CHCl_3_); IR *v_max_* (film): 3402, 3079, 2928, 1716, 1637, 1449, 903, 756; ^1^H-NMR: δ 5.72 (dd, *J* = 17.5 Hz and 10.8 Hz, 2H, H-15); 4.87 (dd, *J* = 10.8 Hz and 1.4 Hz, 1H, H-16) and 4.83 (dd, *J* = 17.5 Hz and 1.4 Hz, 1H, H-16′); 3.42 (d, *J* = 10.9 Hz, 1H, H-19) and 3.14 (d *J* = 10.9 Hz, 1H, H-19′); 0.97 s (3H, H-17); 0.94 s (3H, H-18); 0.78 s (3H, H-20); GC-MS: Rt 15.77-15.83 min; C_20_H_32_O (288); *m/z* 288 (21), 273 (43), 257 (61), 187 (39), 161 (49), 119 (58), 105 (91), 91 (100), 81 (78), 55 (85).

*Sandaracopimaric acid* (**5**). [α]_D_^20^: +16.9 (*c* 0.21, CHCl_3_); IR *v_max_* (film): 3387, 2926, 1691, 1457, 760; ^1^H-NMR: δ 5.75 (dd, *J* = 17.4 Hz and 10.6 Hz, 1H, H-15); 5.20 (s, 1H, H-14); 4.88 (dd, *J* = 17.5 Hz and 1.4 Hz, 1H, H-16) and 4.85 (dd, *J* = 10.8 Hz and 1.4 Hz, 1H, H-16′); 1.18 (s, 3H, H-18); 1.02 (s, 3H, H-17); 0.81 (s, 3H, H-20).

*Sandaracopimaric acid methyl ester* (**5a**). GC-MS: Rt 16.15 min; C_21_H_32_O_2_ (316); *m/z* 316 (3), 301 (10), 257 (10), 180 (10), 121 (100).

*Labda-8(17),14-dien-13-ol* (manool, **6**). [α]_D_^20^: +66.4 (*c* 0.42, CHCl_3_); IR *v_max_* (film): 3458, 3075, 2928, 2864, 2836, 1457, 1382, 1234, 907; ^1^H-NMR: δ 5.89 (dd, *J* = 17.4 Hz and 10.8 Hz, 1H, H-14); 5.18 (dd, *J* = 17.4 Hz and 1 Hz, 1H, H-15) and 5.03 (dd, *J* = 10.8 Hz and 1 Hz, 1H, H-15′); 4.79 (d, *J* = 1 Hz, 1H, H-17) and 4.49 (br s, 1H, H-17′); 1.25 (s, 3H, H-16); 0.84 (s, 3H, H-19); 0.77 (s, 3H, H-18); 0.65 (s, 3H, H-20); GC-MS: Rt 14.19 min, 272 (M^+^-H_2_O) (11), 257 (34), 137 (53), 95 (87), 81 (100), 55 (73).

*18-Hydroxymanool* (**7**). ^1^H-NMR: δ 5.73 (dd, *J* = 17.4 Hz and 10.7 Hz, 1H, H-14); 4.86 (dd, *J* = 10.7 Hz, 1.2 Hz, 1H, H-15) and 4.82 (dd, *J* = 17.4 Hz and 1.2 Hz, 1H, H-15′); 4.79 (s, 1H, H-17) and 4.49 (s, 1H, H-17′); 3.41 (d, *J* = 10.9 Hz, 1 H, H-19) and 3.14 (d, *J* = 10.9 Hz, 1H, H-19′); 1.25 (s, 3H, H-16); 0.94 (s, 3H, H-18); 0.84 (s, 3H, H-20).

*18-Acetoxymanool* (**8**). ^1^H-NMR: δ 5.86 (ddd, *J* = 17.4 Hz, 10.8 Hz and 1.1 Hz, 1 H, H-14); 5.15 (dd, *J* = 17.4 Hz and 1.1 Hz, 1H, H-15) and 5.01 (br d, *J* = 10.8 Hz, 1H, H-15); 4.78 (d, *J* = 1 Hz, 1H, H-17) and 4.49 s (1H, H-17′); 4.17 (d, *J* = 11 Hz, 1H, H-19) and 3.80 (d, *J* = 11 Hz, 1H, H-19′); 1.99 (s, 3H, OAc); 1.23 (s, 3H, H-16); 0.91 (s, 3H, H-18); 0.64 (s, 3H, H-20); EI-MS: C_22_H_36_O_3_ (348) 330 (0.5) (M^+^-18), 270 (3), 255 (10), 189 (10), 147 (16), 135 (38), 93 (37), 79 (44), 43 (100).

*Torulosal* (**9**). ^1^H-NMR: δ 9.69 (s, 1H, H-19); 5.86 (ddd, *J* = 17.4 Hz, 10.8 Hz and 0.6 Hz, 1H, H-14); 5.15 (dd, *J* = 17.4 Hz and 1.1 Hz, 1H, H-15) and 5.01 (br d, *J* = 10.7 Hz, 1H, H-15′); 4.84 (d, *J* = 1 Hz, 1H, H-17) and 4.52 (s, 1H, H-17′); 1.23 (s, 3H, H-16); 0.97 (s, 3H, H-18); 0.52 (s, 3H, H-20). GC-MS: Rt: 16.16 min; C_20_H_32_O_2_ (304); *m/z* 286 (4) (M^+^-18), 257 (21), 218 (9), 189 (15), 147 (32), 133 (39), 119 (50), 107 (62), 93 (68), 81 (100), 55 (91), 43 (99).

*Torulosic acid* (**10**). [α]_D_^20^: +19.1 (*c* 0.22, CHCl_3_). ^1^H-NMR: δ 5.95 (dd, *J* = 17.3 Hz and 10.8 Hz, 1H, H-14); 5.23 (d, *J* = 17.3 Hz, 1H, H-15) and 5.08 (d, *J* = 10.8 Hz, 1H, H-15′); 4.87 (s, 1H, H-17) and 4.55 (s, 1H, H-17′); 1.31 (s, 3H, H-16); 1.23 (s, 3H, H-18); 0.63 (s, 3H, H-20).

*Torulosic acid methyl ester* (**10a**). [α]_D_^20^: +19.8 (*c* 0.50, CHCl_3_); GC-MS: Rt: 16.39 min; 316 (M^+^- H_2_O) (5), 301 (5), 257 (15), 241 (13), 121 (100), 107 (35), 93 (40), 81 (40), 55 (35). ^1^H-NMR: δ 5.90 (dd, *J* = 17.3 Hz and 10.8 Hz, 1H, H-14); 5.21 (d, *J* = 17.3 Hz, 1H, H-15) and 5.05 (d, *J* = 10.8 Hz, 1H, H-15′); 4.87 (s, 1H, H-17) and 4.52 (s, 1H, H-17′); 3.63 (s, 3H, OMe); 1.25 (s, 3H, H-16); 1.18 (s, 3H, H-18); 0.48 (s, 3H, H-20).

*8(17),12,14-Labdatriene* (**11**). GC-MS: Rt 14.15 min; C_20_H_32_ (272); *m/z* 272 (12), 257 (38), 175 (7), 161 (18), 137 (55), 109 (54), 95 (91), 81 (100), 69 (85), 55 (89). ^1^H-NMR: δ 6.28 (dd, *J* = 17.3 Hz and 10.8 Hz, 1H, H-14); 5.36 (t, *J* = 6.3 Hz, 1H, H-12); 5.00 (d, *J* = 17.3 Hz, 1H, H-15) and 4.84 (d, *J* = 10.8 Hz, 1H, H-15′); 4.80 (s, 1H, H-17) and 4.42 (s, 1H, H-17′); 2.37 (br d, *J* = 12.4 Hz, 2 H, H-11); 1.71 (s, 3H, H-16); 0.85 (s, 3H, H-19); 0.78 (s, 3H, H-18); 0.65 (s, 3H, H-20).

*(E)*-*Communic acid* (**12**). ^1^H-NMR: δ 6.30 (dd, *J* = 17.3 Hz and 10.8 Hz, 1H, H-14); 5.39 (t, *J* = 6.3 Hz, 1H, H-12); 5.02 (d, *J* = 17.3 Hz, 1H, H-15) and 4.86 (d, *J* = 10.8 Hz, 1H, H-15′); 4.82 (s, 1H, H-17) and 4.44 (s, 1H, H-17′); 2.38 (br d, *J* = 12.4 Hz, 2 H, H-11); 1.73 (s, 3H, H-16); 1.23 (s, 3H, H-18); 0.63 (s, 3H, H-20).

*(E)*-*Communic acid methyl ester* (**12a**). [α]_D_^20^: +37.17 (*c* 0.53, CHCl_3_); IR *v_max_* (film): 3402, 2932, 2844, 1692; GC-MS: Rt 16.08 min; C_21_H_32_O_2_ (316); *m/z* 316 (19), 301 (13), 257 (19), 241 (20), 175 (53), 133 (40), 121 (100), 119 (66), 105 (56), 93 (63), 79 (73).

*(Z)*-*Communic acid* (**13**). ^1^H-NMR: δ 6.77 (dd, *J* = 17.3 Hz and 10.8 Hz, 1 H, H-14); 5.26 (t, *J* = 6.3 Hz, 1H, H-12); 5.15 (d, *J* = 17.3 Hz, 1H, H-15) and 5.06 (d, *J* = 10.8 Hz, 1H, H-15′); 4.82 (s, 1H, H-17) and 4.47 (s, 1H, H-17′); 1.75 (s, 3H, H-16); 1.23 (s, 3H, H-18); 0.63 (s, 3H, H-20).

*(Z)*-*Communic acid methyl ester* (**13a**). GC-MS: Rt 15.56–15.72 min; C_21_H_32_O_2_ (316); *m/z* 316 (19), 301 (4), 257 (8), 241 (8), 175 (20), 135 (50), 121 (100), 107 (55), 91 (56), 79 (56).

*12-Oxolabda-8(17),13E-dien-19 oic acid* (**14**). [α]_D_^20^: +36.3 (*c* 0.38, CHCl_3_); IR *v_max_* (film): 3455, 2935, 1726, 1671, 1643, 1452, 1232, 1156, 773; ^1^H-NMR: δ 6.77 (dq, *J* = 6.9 Hz and 1.1 Hz, 1H, H-14); 4.67 (s, 1H, H-17) and 4.23 (s, 1H, H-17′); 3.60 (s, 3H, OMe); 2.94 (dd, *J* = 17.5 Hz and 10.9 Hz, 1H, H-11) and 2.50 (dd, *J* = 17.5 Hz and 3 Hz, 1H, H-11′); 2.15 (br d, *J* = 13.3 Hz, 1H, H-3); 1.84 (dd, *J* = 6.9 Hz and 1.1 Hz, 3H, H-15); 1.74 (s, 3H, H-16); 1.18 (s, 3H, H-18); 0.54 (s, 3H, H-20); HR-MS (*m/z*): 318.2210. Calcd for C_20_H_30_O_3_: 318.2195.

*12-Oxolabda-8(17),13E-dien-19 oic acid methyl ester* (**14a**). [α]_D_^20^: +21.68 (*c* 0.14, CHCl_3_); IR *v_max_* (film): 3426, 3075, 2936, 1724, 1672, 1154, 883. GC-MS: Rt 17.66 min; C_21_H_32_O_3_ (332); *m/z* 332 (1), 317 (1), 272 (2), 257 (1), 234 (8), 175 (11), 159 (7), 121 (26), 83 (100), 55 (49).

*Ferruginol* (**15**). GC-MS: Rt 16.74 min; C_20_H_30_O (286); *m/z* 286 (78), 271 (99), 201 (58), 189 (100), 175 (97), 159 (28), 149 (40), 69 (99); ^1^H-NMR: δ 6.85 (s, 1H, H-14); 6.65 (s, 1H, H-11); 3.18 (m, 1H, H-15); 1.24 (d, *J* = 7.0 Hz, 3H, H-16); 1.20 (d, *J* = 7.0 Hz, 3H, H-17); 1.19 (s, 3H, H-18); 0.96 (s, 3H, H-19); 0.93 (s, 3H, H-20).

*Dehydroabietic acid methyl ester* (**16a**). GC-MS: Rt 15.52 min; C_21_H_32_O_2_ (316); *m/z* 316 (22), 301 (22), 257 (38), 241 (100), 173 (29), 212 (61), 195 (69), 81 (72). ^1^H-NMR: δ 7.32 (d, *J* = 8.3 Hz, 1H, H-11); 7.15 (dd, *J* = 8.3 Hz and 1.7 Hz, 1H, H-12); 7.04 (d, *J* = 1.7 Hz, 1H, H-14); 3.80 (s, 3H, OMe); 2.98 (m, 1H, H-15); 1.45 (s, 3H, H-18); 1.38 (d, *J* = 7.0 Hz, 6H, H-16 and H-17); 1.37 (s, 3H, H-20).

*7-Oxoferruginol* *(sugiol)* (**17**). GC-MS: Rt 20.70 min; C_20_H_28_O_2_ (300); *m/z* 300 (70), 285 (80), 243 (30), 215 (46), 203 (49), 69 (72), 44 (100).

## 4. Conclusions

The composition of *A. chilensis* resin was established by spectroscopic and spectrometric means. A GC-MS method was developed for the fast identification of the diterpene constituents in the resin, setting the conditions for new studies on the physiological response of the tree under different environmental stimuli.
